# L-Glutamate Supplementation Improves Small Intestinal Architecture and Enhances the Expressions of Jejunal Mucosa Amino Acid Receptors and Transporters in Weaning Piglets

**DOI:** 10.1371/journal.pone.0111950

**Published:** 2014-11-04

**Authors:** Meng Lin, Bolin Zhang, Changning Yu, Jiaolong Li, Lin Zhang, Hui Sun, Feng Gao, Guanghong Zhou

**Affiliations:** 1 College of Animal Science and Technology, Key Laboratory of Animal Origin Food Production and Safety Guarantee of Jiangsu Province, Synergetic Innovation Center of Food Safety and Nutrition, Nanjing Agricultural University, Nanjing, Jiangsu, China; 2 College of Animal Science and Technology, Jilin Agricultural University, Changchun, Jilin, China; Institute of Biochemistry and Biotechnology, Taiwan

## Abstract

L-Glutamate is a major oxidative fuel for the small intestine. However, few studies have demonstrated the effect of L-glutamate on the intestinal architecture and signaling of amino acids in the small intestine. The aim of this study was to investigate the effects of dietary L-glutamate supplementation on the intestinal architecture and expressions of jejunal mucosa amino acid receptors and transporters in weaning piglets. A total of 120 weaning piglets aged 35±1 days with an average body weight at 8.91±0.45 kg were randomly allocated to two treatments with six replicates of ten piglets each, fed with diets containing 1.21% alanine, or 2% L-glutamate. L-Glutamate supplementation increased the activity of glutamate oxaloacetate transaminase (GOT) in the jejunal mucosa. Also, the mRNA expression level of jejunal mucosa glutamine synthetase (GS) was increased by L-glutamate supplementation. The height of villi in duodenal and jejunal segments, and the relative mRNA expression of occludin and zonula occludens protein-1 (ZO-1) in jejunal mucosa were increased by dietary L-glutamate supplementation. L-Glutamate supplementation increased plasma concentrations of glutamate, arginine, histidine, isoleucine, leucine, methionine, phenylalanine and threonine. L-Glutamate supplementation also increased the relative mRNA expression of the jejunal mucosa Ca^2+^-sensing receptor (CaR), metabotropic glutamate receptor 1 (mGluR1) and metabotropic glutamate receptor 4 (mGluR4), and neutral amino acid transporter B^0^-like (SLC1A5) in the jejunal mucosa. These findings suggest that dietary addition of 2% L-glutamate improves the intestinal integrity and influences the expression of amino acid receptors and transporters in the jejunum of weaning, which is beneficial for the improvement of jejunal nutrients for digestion and absorption.

## Introduction

L-Glutamate is one of the most common amino acid in animal and plant proteins as well as in milk [1]–[3]. L-Glutamate is also a functional amino acid in cell metabolism and signaling [4]. Emerging evidence shows that L-glutamate is a critical oxidative substrate for the intestinal mucosa [5], [6] and a precursor of important molecules, such as glutathione and the polyglutamated folate cofactors [7], [8]. These facts suggest that dietary glutamate may be a key factor for the maintenance of mucosal health. Tracer studies in piglets and rats have confirmed that dietary glutamate is catabolized almost completely in the small intestinal to yield ATP and CO_2_ [9]. The intestinal epithelial cells have high ATP production and utilization, which is in rapid renewal and responsible for the nutrient absorption process [6], [10]. Therefore, dietary L-glutamate supplementation may improve intestinal health in weaning piglets.

In addition, besides its nutritional role, L-glutamate is an important excitatory neurotransmitter in the body. Some studies have demonstrated that multiple glutamate receptors and transporters have been found in the gastrointestinal tract [11]–[13]. Importantly, emerging evidence also showed that oral administration of monosodium glutamate increased the expression of glutamate receptors and transporters in the gastrointestinal tract of young piglets [14]. In other studies, luminal L-glutamate was shown to enhance duodenal mucosal defense mechanisms via multiple G protein-coupled receptors (GPCRs), including the taste receptor type 1, member 1 and the taste receptor type 1, member 3 (T1R1/T1R3) heterodimer, and the metabotropic glutamate receptors (mGluR) [15], [16]. Thus, glutamate is a functional amino acid which beneficially enhances nutrient sensing and transport in the gastrointestinal tract. Generally, the gut is the primary organ that is involved in digestion, absorption and metabolism of dietary nutrients. The important process for absorption of free amino acid is mainly mediated by specific transporter in the jejunum [17], [18]. The different amino acid transporters have been identified as the major intestinal transporters for neutral, basic, and acidic amino acid, such as solute carrier family 7, member 7 (SLC7A7), neutral amino acid transporter B^0^-like (SLC1A5), solute carrier family 7, member 9 (SLC7A9), solute carrier family 6, member 19 (SLC6A19) and solute carrier family 1, member 1 (SLC1A1) [17]. Previous reports have indicated that the importance of amino acid in the small intestine is to maintain homeostasis of overall protein nutrition in body [19]. However, the effects of L-glutamate supplementation on the absorption of amino acids in the small intestine of weaning piglets are still unknown. We hypothesized that dietary supplementation with L-glutamate may modulate the mRNA expression of amino acid receptors and transporters, which are associated with the absorption in the small intestine of weaning piglets.

Based on the foregoing, the purpose of the present study was to evaluate the effects of L-glutamate supplementation on the intestinal architecture, expressions of amino acid receptors, transporters and intestinal tight proteins in the jejunum of weaning piglets.

## Materials and Methods

### Animals and experimental design

All the procedures including animal care and experiment treatments in the present study were approved by the Institutional Animal Care and Use Committee of Nanjing Agricultural University. A total of 120 crossbred males (castrated, at 10 days of the age) piglets aged 35±1 days with an average body weight of 8.91±0.45 kg were randomly allocated to two treatment groups. Each treatment group had six pens of ten piglets. The diets were formulated with 1.21% alanine or 2% L-glutamate on the basis of maize-soybean- based diets at the expense of the same amount of maize. The dosage of L-glutamate supplementation and alanine as isonitrogenous control were according to the previous studies [20]–[22]. Alanine and L-glutamate were obtained from Tiancheng Pharmaceutical Co. (Tianjin, China). Diets were formulated to meet or exceed requirements suggested by the National Research Council (NRC 2012) [23] (**Table 1**). The basal diets contained 19.96% crude protein and 2.62% glutamate, which were analyzed as previously described [1]. During the entire experimental period, piglets were allowed *ad libitum* access to feed and water. The room temperature was maintained at 25–27°C. All the piglets were fed three times per day at 06.30, 11.00, and 18.00 hours.

**Table 1:** Ingredients and nutrient content of the basal diets of weaning piglets.

Ingredients	(%)	Nutrient content (%)	
Maize	49.50	Crude protein ‡	19.96
Wheat flour	11.06	Crude fat ‡	17.57
Soybean meal	19.30	Crude fiber	2.00
Expanded soybean	9.50	Ash ‡	6.44
Whey powder	5.00	Net energy(MJ/kg)	10.13
Fish meal	2.50	Lys	1.24
DL-Methionine	0.11	Met + Cys	0.69
Lysine-HCl	0.37	Thr	0.74
Threonine	0.11	Trp	0.21
Dicalcium phosphate	0.62	Arg	1.15
Limestone	0.63	His	0.43
Salt	0.30	Ile	0.71
Vitamin and mineral premix†	1.00	Leu	1.41
		Phe	0.81
		Val	0.78
		Ca	0.70
		Available phosphorus	0.33

† Premix per kg diet provided: iron 150 mg; copper 171.5 mg; zinc 109.5 mg; manganese 32 mg; selenium 0.45 mg; iodine 0.40 mg; choline, 500 mg; retinyl acetate, 11000 IU; cholecalciferol, 2000 IU; DL-α-tocopheryl acetate, 30 IU; menadione sodium bisulphite, 4.4 mg; thiamin mononitrate, 1.5 mg; riboflavin, 6 mg; pyridoxine hydrochloride, 3 mg; cyanocobalamin, 3.2 mg; D-pantothenic acid, 15 mg; nicotinic acid, 33 mg; D-biotin, 0.20 mg; folic acid, 1.65 mg.

‡ Nutrient content of the diets were the value of measurement.

### Collection of samples

At 63 days of the age, 2 h after the last meal, six piglets were randomly selected from each treatment (one pig from each pen) based on average body weight for sampling. Blood samples (10 mL) were obtained from the jugular vein into heparinized tubes, followed immediately by centrifugation at 3000 g for 10 min at 4°C. The supernatant fluid (plasma) was collected and immediately stored at −20°C for amino acids analysis. Immediately after blood sampling, piglets were anaesthetized with an intravenous injection of sodium pentobarbital (50 mg/kg BW) and bled by exsanguinations. The entire small intestine was then immediately collected and flushed with pre-cooled saline, to remove their contents. Intestinal mucosa was scraped from the underlying musculature with a glass microscope slide and was immediately frozen in liquid nitrogen and stored at −80°C until required for further analysis. Segments of duodenum, jejunum, and ileum (∼3 cm in length for each segment) were fixed in 4% paraformaldehyde for subsequent morphometric analysis.

### Determination of glutamate oxaloacetate transaminase (GOT) and glutamate pyruvate transaminase (GPT) activities in jejunal mucosa

The activities of GOT and GPT in the jejunal mucosa were determined using commercially available kits (Nanjing Jiancheng Bioengineering Institute, Nanjing China) according to the manufacturer's instruction. Each treatment had six independent piglet samples. The assays were performed in triplicate for each sample, and the mean values of each samples was calculated for statistical.

### Determination of plasma amino acids

Amino acids (AA) were determined following acid hydrolysis using a Hitachi L-8900 amino acid analyzer (Hitachi, Tokyo, Japan). Plasma AA contents were determined as previously described [24]. Briefly, 1 mL of plasma and 2.5 mL of 7.5% trichloroacetic acid were mixed thoroughly and centrifuged at 12,000×g at 4°C for 15 min. The supernatant was filtered through a 0.45 µm membrane and then analyzed for AA using an ion-exchange AA analyzer (Hitachi).

### Examination of small-intestinal architecture

Paraformaldehyde-fixed duodenum, jejunum and ileum samples (3 cm) were embedded in paraffin and cut approximately 5 µm thick using a microtome and then stained with hematoxylin and eosin. In each section, villus height (determined as the distance from the villus tip to the crypt mouth) and their associated crypt depth (measured from the crypt mouth to the base) were measured using a light microscope (Nikon, Japan) with a computer-assisted morphometric system. The means of measurements was calculated to yield three values per piglet. These procedures were also processed by an observer unaware of the dietary treatments.

### RNA extraction and cDNA synthesis

Jejunal mucosa sample was pulverized in liquid nitrogen. Total RNA was extracted from tissue samples using the TRIzol reagent (Invitrogen Company, Carlsbad, CA, USA) and treated with DNase I (Invitrogen Company) according to the manufacturer's guidelines. RNA was reverse transcribed to complementary DNA (cDNA) using a PrimeScript 1st Strand cDNA Synthesis Kit (Takara, Ostu, Japan) according to the manufacturer's protocol. Primers were designed with software (Oligo 7.0; Molecular Biology Insights, Cascade, CO) according to the gene sequence of pig to produce an amplification product (**Table 2**).

**Table 2:** Primer pairs used in the RT-PCR.

Genes	Accession no.	Primers	Sequences (5′→3′)
GLUD1	NM_001244501.1	Forward	ACCCACAGCAGAGTTCCAAG
		Reverse	TCAGGTCCAGTCCCAGGTTA
GS	AY216477.1	Forward	AGTGTGTTAGTGGGGAGGGA
		Reverse	GCCATCCATTTACGCCGAAC
Occludin	NM_001163647.2	Forward	GCTTTGGTGGCTATGGAAGT
		Reverse	CCAGGAAGAATCCCTTTGCT
ZO-1	XM_005659811.1	Forward	GAGTTTGATAGTGGCGTT
		Reverse	GTGGGAGGATGCTGTTGT
Claudin-1	NM_001244539.1	Forward	GCCCTACTTTGCTGCTCCT
		Reverse	TTCTGGTTGTTCCCACACG
CaR	NM_001278748	Forward	TGCCCAGATGACTTCTGGTCC
		Reverse	GCACGAGATGCAGAGCACGAAGC
T1R1	XM_003356140	Forward	TCCCTGGGCTTCATACTGG
		Reverse	TTCTCTGGCAAGTCCTTACCC
T1R3	NM_001113288	Forward	AGGAAATCAACAACGGATCG
		Reverse	CTGCGTGTAGTCGCAGTAGG
mGluR1	XM_005659163.1	Forward	GAAGTGATGGATGGGCAGAC
		Reverse	AACTCAGGGAACCAGGGATT
mGluR4	XM_005665894.1	Forward	CAAGACCAACCGCATCTACC
		Reverse	GTCCACCACAAACCACACG
SLC1A1	NM_001164649	Forward	CAAACTGGGCCTTTAACTGG
		Reverse	TGTTGCTGAACTGGAGGAGA
SLC6A19	XM_003359855	Forward	CACAACAACTGCGAGAAGGA
		Reverse	CCGTTGATAAGCGTCAGGAT
SLC1A5	XM_003355984	Forward	GCCAGCAAGATTGTGGAGAT
		Reverse	GAGCTGGATGAGGTTCCAAA
SLC7A9	NM_001110171	Forward	GAACCCAAGACCACAAATC
		Reverse	ACCCAGTGTCGCAAGAAT
SLC7A7	NM_001110421	Forward	AGGAGAACCCACAGATTAGC
		Reverse	GCGGAGGAGGAGAAGAA
β-actin	XM_003357928	Forward	ATGCTTCTAGACGGACTGCG
		Reverse	GTTTCAGGAGGCTGGCATGA

All these primer sequences were designed based on the accession numbers described above.

*GLUD1* glutamate dehydrogenase 1, *GS* glutamine Synthetase, *ZO-1* zonula occludens protein-1, *CaR* Ca^2+^-sensing receptor, *T1R1* taste receptor type 1 member 1, *T1R3* taste receptor type 1 member 3, *mGluR1* metabotropic glutamate receptor 1, *mGluR4* metabotropic glutamate receptor 4, *SLC1A1* solute carrier family 1 (neuronal/epithelial high affinity glutamate transporter, system Xag), member 1, *SLC6A19* solute carrier family 6 (neutral amino acid transporter), member 19, *SLC1A5* neutral amino acid transporter B^0^-like, *SLC7A9* solute carrier family 7 (glycoprotein-associated amino acid transporter light chain, b^o,+^ system), member 9, *SLC7A7* solute carrier family 7 (amino acid transporter light chain, y^+^L system), member 7.

### Quantification of mRNA levels

The yield and quality of the RNAs were checked spectrophotometrically using OD_260_ and OD_280_ measurements (ND-100, NanoDrop Technologies, Rockland, DE). All samples had an OD_260_/OD_280_ ratios between 1.8 and 2.0. The integrity of the RNA preparations was verified by visualization of the 18 S and 28 S ribosomal bands stained with ethidium bromide after electrophoresis on 2% agarose gels (E-gel; Invitrogen Inc., Carlsbad, CA). Real time RT-PCR (SYBR Premix Ex Taq, catalogue no. RR420A) was performed with a total volume of 20 µL system containing 10 µL SYBR Premix Ex Taq (2×), 0.4 µL ROX Reference Dye (50×), 2.0 µL cDNA and 0.4 µL each of forward and reverse primers according to the instructions of manufacturer. PCR program consisted of one cycle at 95°C for 30 s, 40 cycles at 95°C for 5 s and 60°C for 34 s. A melting curve was conducted to verify the specificity of PCR amplified product. The mRNA abundance values for each sample were normalized using β-actin as an internal control according to the 2^−ΔΔCT^ method [25].

### Statistical Analysis

Data are expressed as means with SEM. Statistical analysis of all data was performed by independent-sample T-test with SPSS software (16.0, SPSS Inc., Chicago, USA). The individual piglet was considered the experimental unit for intestinal architecture, plasma amino acids and gene expression. All statements of significance were based on a probability of less than 0.05.

## Results

### GOT and GPT activities in jejunal mucosa

The activities of GOT and GPT in the jejunal mucosa of weaning piglets are shown in **Table 3**. Compared with the control group, dietary L-glutamate supplementation increased (*P*<0.05) the activity of GOT in the jejunal mucosa. The activity of GPT in the jejunal mucosa did not differ between the groups.

**Table 3:** Effects of dietary L-glutamate supplementation on jejunal mucosa GOT and GPT in weaning piglets (n = 6).

Items	Dietary treatments		SEM	P value
	Ala	Glu		
GOT (U/g of protein)	46.08	55.73	2.21	0.02
GPT (U/g of protein)	11.76	12.46	0.91	0.72

*GOT*, glutamate oxaloacetate transaminase, *GPT* glutamate pyruvate transaminase.

*Ala* alanine, *Glu* L-glutamate. *SEM* standard error of the means.

*Ala* was used as the control of isonitrogen balance.

### Relative mRNA expression of jejunal mucosa glutamine synthetase (GS) and glutamine synthetase (GLUD1)

**Figure 1:**
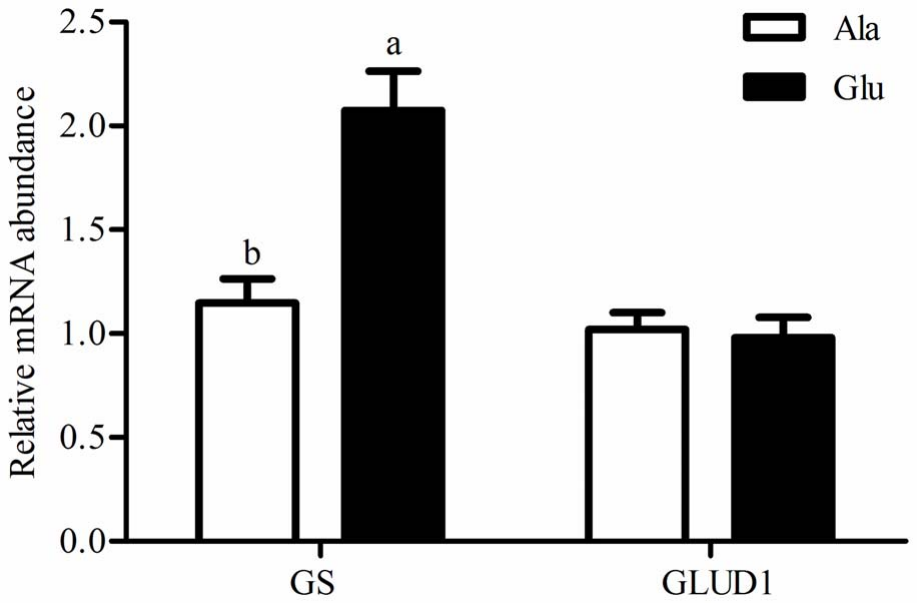
Relative mRNA expression of jejunal moucosa glutamine synthetase (GS) and glutamate dehydrogenase 1(GLUD1). Ala, diet supplemented with 12.1 g alanine/kg, Glu, diet supplemented with 20 g glutamate/kg. mRNA expression levels were normalized using β-actin as an internal control. Values are expressed as mean±SEM. ^a, b^Mean values within different letters were significantly different (P<0.05).

The abundances of GS and GLUD1 mRNAs in the jejunal mucosa of weaning piglets are shown in **Fig. 1**. Dietary L-glutamate supplementation increased the mRNA expression of jejunal mucosa GS (*P*<0.05), but did not affect the mRNA expression of jejunal mucosa GLUD1.

### Concentrations of amino acids in plasma

The concentrations of plasma amino acids in weaning piglets are shown in **Table 4**. Compared with the control group, dietary L-glutamate supplementation increased (*P*<0.05) the concentrations of arginine, histidine, isoleucine, leucine, methionine, phenylalanine, threonine valine, alanine, glutamate, glyine, proline, serine tyrosine and ornithine in the plasma.

**Table 4:** Effects of dietary L-glutamate supplementation on intestinal morphology in weaning piglets (n = 6).

Items	Dietary treatments		SEM	P value
	Ala	Glu		
*Duodenum*				
Villus height (µm)	367.94	390.87	3.89	0.002
Crypt depth (µm)	224.69	240.95	3.93	0.037
Villus height: Crypt depth	1.65	1.63	0.02	0.762
*Jejunum*				
Villus height (µm)	355.10	376.23	4.45	0.015
Crypt depth (µm)	160.05	171.01	3.26	0.093
Villus height: Crypt depth	2.24	2.21	0.03	0.698
*Ileum*				
Villus height (µm)	309.04	304.67	3.19	0.502
Crypt depth (µm)	181.01	183.03	4.88	0.839
Villus height: Crypt depth	1.75	1.70	0.04	0.595

*Ala* alanine, *Glu* L-glutamate, *SEM*, standard error of the mean.

*Ala* was used as the control of isonitrogen balance.

### Small intestinal architecture

**Table 5** shows the small intestinal architecture of weaning piglets. Compared with the control, dietary L-glutamate addition significantly increased (*P*<0.05) duodenal villus height and crypt depth. Moreover, the jejunal villus height was also significantly increased (*P*<0.05) compared with the control. Feeding L-glutamate to weaning piglets did not significantly affected villus height or crypt depth in the ileum. Interestingly, compared with the control, there were no significant differences in the ratios of villus height or crypt depth in the entire small intestine.

**Table 5:** Effect of dietary L-glutamate supplementation on the concentrations plasma amino acid in weaning piglets (n = 6).

Amino acid	Dietary treatments		SEM	P value
	Ala	Glu		
Arg	238.43	312.99	12.99	<0.001
His	164.03	244.67	16.41	0.006
Ile	131.55	180.57	9.52	0.003
Leu	138.29	201.03	11.24	0.001
Lys	132.45	167.97	7.94	0.016
Met	167.20	243.63	15.68	0.006
Phe	102.24	134.34	7.20	0.017
Thr	143.40	189.77	14.05	0.100
Val	129.48	173.49	8.22	0.002
Ala	197.93	234.96	12.03	0.130
Asp	22.77	22.68	1.03	0.967
Cys	374.23	382.74	14.30	0.782
Glu	110.00	164.58	10.39	0.002
Gly	192.55	236.21	11.05	0.041
Pro	219.88	234.24	8.24	0.410
Ser	175.56	232.54	11.41	0.005
Tyr	145.28	214.48	12.53	0.001
Orn	131.83	172.07	6.73	<0.001
Cit	160.08	186.13	4.76	0.001

*Ala* alanine, *Glu* L-glutamate, *SEM* standard error of the mean.

The unit of amino acid concentratin was nmoL/Ml.

*Ala* was used as the control of isonitrogen balance.

### Relative mRNA expression of jejunal mucosa occludin, zonula occludens protein-1 (ZO-1) and claudin-1

**Figure 2:**
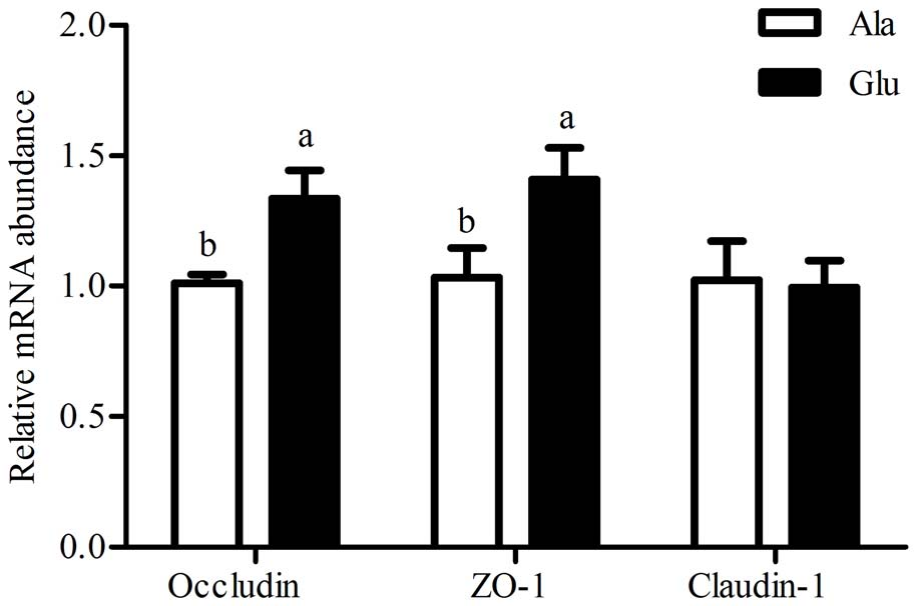
Relative mRNA expression of jejunal mucosa occludin, zonula occludens protein-1 (ZO-1), and claudin-1. Ala, diet supplemented with 12.1 g alanine/kg, Glu, diet supplemented with 20 g glutamate/kg. mRNA expression levels were normalised using β-actin as an internal control. Values are expressed as mean ± SEM. ^a, b^Mean values within different letters were significantly different (P<0.05).

The abundance of occludin, ZO-1 and claudin-1 in the jejunal mucosa are shown in **Fig. 2**. Compared with the control, dietary L-glutamate supplementation significantly increased (*P*<0.05) mRNA expression of occludin and ZO-1 in the jejunal mucosa. However, L-glutamate supplementation in the diet of weaning piglets did not affect claudin-1 expression in the jejunum mucosa.

### Relative mRNA expression of jejunal mucosa amino acid receptors and transporters

The abundance of amino acids (CaR, T1R1, T1R3, mGluR1 and mGluR4) and transporters (SLC1A5, SLC7A9 SLC6A19, SLC7A7 and SLC1A1) mRNAs in the jejunal mucosa of weaning piglets are shown in **Table 6**. Compared with the control, dietary L-glutamate supplementation significantly increased (*P*<0.05) mRNA expression of CaR, mGluR1, mGluR4 and SLC1A5 in the jejunum mucosa. No significant differences in the mRNA expression of T1R1, T1R3, SLC7A9 SLC6A19, SLC7A7 and SLC1A1 were observed between treatments.

**Table 6:** Effect of dietary L-glutamate supplementation on Relative mRNA expression of jejunal mucosa amino acid receptors and transporters in weaning piglets (n = 6).

Items	Dietary treatments		SEM	P value
	Ala	Glu		
*Receptors*				
CaR	1.19	1.63	0.10	0.017
T1R1	1.16	1.20	0.11	0.870
T1R3	1.06	1.04	0.09	0.885
mGluR1	0.84	1.23	0.10	0.031
mGluR4	0.97	1.28	0.08	0.046
*Transporters*				
SLC6A19	1.14	3.44	0.35	<0.001
SLC7A9	1.26	1.14	0.07	0.399
SLC1A5	1.02	0.91	0.05	0.346
SLC7A7	1.13	1.02	0.06	0.447
SLC1A1	1.19	1.01	0.06	0.774

*Ala* alanine, *Glu* L-glutamate, *SEM* standard error of the mean.

*Ala* was used as the control of isonitrogen balance.

mRNA expression levels of CaR, T1R1, T1R3, mGluR1, mGluR4, SLC6A19, SLC7A9, SLC1A5, SLC7A7 and SLC1A1 were normalized using β-actin as an internal control.

*CaR* Ca^2+^-sensing receptor, *T1R1* taste receptor type 1 member 1, *T1R3* taste receptor type 1 member 3, *mGluR1* metabotropic glutamate receptor 1, *mGluR4* metabotropic glutamate receptor 4, *SLC1A1* solute carrier family 1 (neuronal/epithelial high affinity glutamate transporter, system Xag), member 1, *SLC6A19* solute carrier family 6 (neutral amino acid transporter), member 19, *SLC1A5* neutral amino acid transporter B^0^-like, *SLC7A9* solute carrier family 7 (glycoprotein-associated amino acid transporter light chain, b^o,+^ system), member 9, *SLC7A7* solute carrier family 7 (amino acid transporter light chain, y^+^L system), member 7.

## Discussion

Previous studies in piglets and preterm infants have shown that dietary L-glutamate is the most important contributor to mucosal oxidative metabolism [4]–[6]. L-Glutamate catabolism occurs in the intestinal enterocyte by GOT, GPT and GLUD1 to produce L-alanine, L-aspartate and α-ketoglutarate [26]. α-Ketoglutarate produced by transamination can then enter the mitochondria, and its metabolism via the tricarboxylic acid (TCA) cycle produces reduced coenzymes (NADH, FADH2) used by the mitochondria for ATP synthesis. The intestinal epithelial cells have high ATP utilization, which is in rapid renewal and responsible for the nutrient absorption process [6], [10]. In this study, L-glutamate supplementation beneficially increased the activity of GOT and the mRNA expression of jejunal mucosa GS (Table 3; Fig. 1). These findings suggest that L-glutamate supplementation may increase the content of glutamine, α-ketoglutarate and L-aspartate in the jejunal mucosa of weaning piglets. L-Aspartate produced by L-glutamate transamination can enter mitochondria and can also be oxidized in the TCA cycle, thus representing another oxidative fuel for enterocytes [27]. Therefore, the central importance of L-glutamate as an oxidative fuel is that it can improve digestion and absorption function of weaning piglets.

The small intestine of weaning piglets is likely to be damaged if it cannot obtain sufficient nutrients to meet the demands for mucosal protein synthesis and growth. The results of the macroscopic observation and architecture evaluation of the small intestine further demonstrated that dietary L-glutamate supplementation improved intestinal abnormalities (Table 4). In accordance, a recent study has reported that dietary supplementation 1% to 4% monosodium glutamate increased jejunal villus height and crypt depth in weanling piglets [21]. More recently, Xiao et al., [28] found that glutamate supplementation could significantly prevent intestinal mucosal atrophy via promotion of intestine epithelial cell proliferation in total parenteral (TPN) mice. In addition, some previous studies demonstrated that the glutamate is also involved in the mucosal healing process by mediating proliferation of intestinal epithelial cells [7],[29]. Therefore, the improvement of small intestinal morphology in weanling piglets could be associated with the L-glutamate supplementation.

The cells of the intestinal epithelium are linked by several unique proteins, including the transmembrane protein occludin, junctional adhesion molecule [30], members of the claudin family [31], linker proteins such as ZO-1 [32], and others. The foremost and critical components in the structural and functional organ of the tight junctions are occludin, ZO-1 and claudin-1 [33]. Occludin is an integral membrane protein of the epithelial tight junction, having functional importance in maintaining the integrity and barrier function of the tight junction [34]. ZO-1 is an important linker protein in tight junctions and acts as a bridge between the plasma membrane and cytoskeleton proteins [33]. Claudin-1 appears fairly tightly localized to the expression of ZO-1 in the small intestine [35]. To better clarify the molecular mechanism for intestine architecture in weaning piglets fed L-glutamate, we determined the changes in mRNA expression of occludin, ZO-1 and claudin-1 in jejunum mucosa. Our results showed that L-glutamate supplementation significantly increased jejunum mucosa occludin and ZO-1 mRNA expression (Fig. 2). Evidence from animals and cell studies shows that glutamine is important for intestinal barrier function and regulation of tight junction protein [36], [37]. As well, prohibiting the conversion from glutamine to glutamate has been shown inhibit the tight junction protein expression enhance effect of glutamine [38], [39]. Thus, the addition of L-glutamate to diet provided sufficient energy for the jejunum to facilitate the cell-cell contact associated genes, including tight junction protein. In contrast, Xiao et al., [28] showed that L-glutamate supplementation significantly decreased occludin and ZO-1 protein expression in the total parenteral (TPN) mice. The difference if the results could be ascribed to the method of L-glutamate administration (feed intake VS oral administration). More investigations *in vitro* and *in vivo* are needed to further determine the effects of L-glutamate on tight junction protein expression and to clarify the exact role of L-glutamate in epithelial barrier function.

Amino acids are essential nutrients for protein synthesis and other metabolic functions in animals. An interesting observation from this study was that dietary supplementation with L-glutamate increased the plasma concentrations of a number of amino acids (Table 5). These amino acids included arginine, histidine, isoleucine, leucine, methionine, phenylalanine, threonine, valine, alanine, glutamate, glyine, proline, serine, tyrosine and ornithine. In accordance with some previous studies in animals and humans, the intake of L-glutamate increased the concentrations of certain essential amino acids in plasma [21], [40]. A study showed that 30–50% of nutritionally essential amino acids in the diet were degraded by the pig small intestine in the first pass, leading to a reduced efficiency of utilization of dietary protein [5]. It is possible that L-glutamate reduces the catabolism of these amino acids in the small intestine, enhancing their entry into the portal circulation.

The sensing of luminal contents is important in initiating the appropriate response of digestion and absorption of nutrients and plays an important role in host defense mechanisms [41]. It has been shown that the taste-sensing system plays a critical role in luminal sensing in the small intestinal tract, especially for luminal nutrient signals [16], [42]. The interaction between luminal nutrients and taste receptor through G-coupled proteins initiates various downstream pathways and exerts different effects on the body that depends on the combination of 3 taste receptors [43]–[45]. In our study, the higher expression of CaR, mGluR1 and mGluR4 in the jejunal mucosa of the dietary L-glutamate supplemented group (Table 6) was likely resulted from glutamate receptors which detected the ingested glutamate and transmitted this information to intestinal cells. Some studies have indicated that GPCRs, including T1R1/T1R3 and mGluR, regulate intestinal hormone secretion and intestinal nutrient absorption [46], [47]. Therefore, this process indicated that the high expression of glutamate receptor is involved in the jejunal control of protein digestion and absorption. However, changes in the up-regulated expression of glutamate receptors may participate in the intestinal hormone secretion in the piglets, but further experiments are required to test this hypothesis.

Dietary amino acid is absorbed via their transporter systems on the mucosal side of the small intestine. Different amino acid transporters have unique substrate specificities [48]. For example, transporters have been characterized that are specific for basic amino acids (SLC7A7), neutral amino acids (SLC6A19, SLC1A5 and SLC7A9) and acidic amino acids (SLC1A1) [17]. To date, the exact role of L-glutamate in modulating AA transporters expression has yet to be defined by the limited available data. In this research, dietary L-glutamate supplementation increased SLC1A5 mRNA expression in the jejunal mucosa (Table 6). L-Glutamate improved small intestine villi height and enhanced tight junction protein expression. Therefore, L-glutamate regulates the expression of amino acid transporters in the jejunal mucosa of weaning piglets by facilitating the repair of intestinal architecture. In addition, L-glutamate increased small intestine mucosa utilization of certain amino acid, such as arginine, lysine and branched-chain amino acids. Recently, some studies have reported that dietary supplementation with lysine or arginine regulated the intestine expression of amino acid transporters in weanling pig [49]–[51]. Therefore, whether L-glutamate directly or indirectly regulates amino acid transporters in jejunal mucosa is still unknown; further research will be required to elucidate the underlying mechanisms.

In conclusion, dietary L-glutamate supplementation improved intestinal integrity and increased amino acid receptors and transporters, including CaR, mGluR1, mGluR4 and SLC1A5, in the jejunal mucosa of weaning piglets, these changes are beneficial for the improvement of jejunal nutrient digestion and the function absorption process. Therefore, the findings of this study could help advance our knowledge of L-glutamate function but further research will be required to elucidate the underlying mechanisms.
